# 超高效液相色谱-串联质谱法测定人体全血中22种有机磷酸酯及其代谢物和11种邻苯二甲酸酯类代谢物

**DOI:** 10.3724/SP.J.1123.2025.09019

**Published:** 2026-06-08

**Authors:** Feiyun SHI, Haoyu XU, Junhong LI, Mengyuan XU

**Affiliations:** 1.常州市疾病预防控制中心，江苏 常州 213022; 1. Changzhou Center for Disease Control and Prevention，Changzhou 213022，China; 2.常州市中医医院，江苏 常州 213022; 2. Changzhou Hospital of Traditional Chinese Medicine，Changzhou 213022，China

**Keywords:** 有机磷酸酯, 邻苯二甲酸酯类代谢物, 全血, 超高效液相色谱-串联质谱法, organic phosphate esters, phthalate ester metabolites, whole blood, ultra performance liquid chromatography-tandem mass spectrometry （UPLC-MS/MS）

## Abstract

有机磷酸酯（organic phosphate esters， OPEs）和邻苯二甲酸酯（phthalate esters， PAEs）是两类人工合成的化合物，常作为阻燃剂和增塑剂以物理结合的方式被添加到各种工业和消费品中，二者均具有多脏器累积毒性。本研究采用超高效液相色谱-串联质谱（UPLC-MS/MS）建立了全血中22种OPEs及其二酯代谢物（mOPEs）和11种邻苯二甲酸酯类代谢物（mPAEs）的检测方法。全血样本采用HMR S-micro BIO 96孔固相萃取柱净化，使用Ghost Trap DS-HP色谱柱（30 mm×2.1 mm）作为背景鬼峰捕集柱，以ACQUITY UPLC^®^ BEH C18色谱柱（150 mm×2.1 mm，1.7 μm）作为分析柱，采用0.5 mmol/L乙酸铵溶液和甲醇为流动相进行梯度洗脱，质谱采用电喷雾离子源（ESI）电离，以正负离子扫描、多反应监测（MRM）模式检测，同位素内标法定量。结果表明，33种目标化合物在15 min内均可实现基线分离，目标化合物在相应质量浓度范围内线性关系良好（*r*为0.993 3~0.999 8），检出限为0.003～0.31 ng/mL，定量限为0.01～1.02 ng/mL。全血样本加标回收率为60.5%～138.3%，相对标准偏差（RSD）为1.6%～11.0%。应用该方法对14个全血样本进行检测，在14份样本中均检出磷酸三（1-氯-2-丙基）酯、磷酸三苯酯（TPHP）、磷酸2-乙基己基二苯基酯、邻苯二甲酸单（2-乙基-5-羟己基）酯、邻苯二甲酸单正丁酯和邻苯二甲酸单正辛酯，且TPHP的检出量最高（10.04 ng/mL）。该方法具有操作简便、灵敏度高、准确性好、所需样品量少以及节约试剂等优点，适用于人体的生物监测，为人群有机磷酸酯和邻苯二甲酸酯类物质的监测提供了理论依据。

## Abstract (en)

Organic phosphate esters （OPEs） and phthalate esters （PAEs） are two classes of synthetic compounds widely incorporated into various industrial and consumer products as flame retardants and plasticizers. Both exhibit multi-organ cumulative toxicity. In this study， an ultra performance liquid chromatography-tandem mass spectrometry （UPLC-MS/MS） method was developed for the determination of 22 OPEs and their diester metabolites （mOPEs） and 11 phthalate ester metabolites （mPAEs） in whole blood. The whole blood samples were extracted with 0.1% formic acid-acetonitrile by liquid-liquid extraction for protein precipitation， and then purified by HMR S-micro BIO 96-well solid phase extraction column. The filtrate was blown to near dryness with nitrogen， and reconstituted with 0.1% formic acid-acetonitrile solution. The target compounds were detected by UPLC-MS/MS. A Ghost Trap DS-HP chromatographic column （30 mm×2.1 mm） was used as the background ghost peak trap column， and an ACQUITY UPLC^®^ BEH C18 chromatographic column （150 mm×2.1 mm， 1.7 μm） was used as the analytical column. Methanol and 0.5 mmol/L ammonium acetate were used as mobile phase for gradient elution. Electrospray ionization （ESI） was used for MS detection， with both positive and negative ion scanning in the multiple reaction monitoring （MRM） mode. Isotope internal standard method was used for quantification. The results showed that 33 target compounds could be separated within 15 min， and good linear relationships were observed for all target compounds across their respective mass concentration ranges （*r*=0.993 3-0.999 8）. The limits of detection were 0.003-0.31 ng/mL and the limits of quantification were 0.01-1.02 ng/mL. The spiked recoveries of 33 targets in whole blood samples ranged from 60.5% to 138.3%， with relative standard deviations （RSDs） from 1.6% to 11.0%. The detection rates of tris-（2-chloroisopropyl）phosphate （TCiPP）， triphenyl phosphate （TPHP）， 2-ethylhexyl diphenyl （EHDPP）， mono（2-ethyl-5-hydroxyhexyl） phthalate （MEHHP）， mono-*n*-butyl phthalate （MNBP） and mono-*n*-octyl phthalate （MOP） were 100% in 14 whole blood samples， and the highest detection amount of TPHP was 10.04 ng/mL. The developed method is simple to operate， sensitive， accurate， requires small sample volumes， and is cost-effective on reagents. It is suitable for human biomonitoring， and provides a theoretical basis for monitoring organic phosphate and phthalic ester substances in the population.

有机磷酸酯（OPEs）和邻苯二甲酸酯（PAEs）是人工合成的两大类化学物质，被广泛添加到各种工业和消费品中，主要用作阻燃剂和增塑剂^［1，2］^。OPEs是新型阻燃剂的代表物质，已有报道超过40种OPEs作为阻燃剂添加到日常消费品中，包括电子设备、家居装饰、食品包装、纺织品和工程塑料等^［3］^。动物实验表明OPEs暴露会产生肝脏毒性、神经毒性、发育毒性、生殖毒性、致癌性和内分泌破坏作用，已被欧盟列为高度关注物质^［4，5］^。随着OPEs的毒性作用越来越受到关注，有研究表明部分OPEs在哺乳动物体内通过羟基化和氧化与葡萄糖醛酸酶、谷胱甘肽酶等偶联，形成OPEs二酯代谢产物（mOPEs），这些mOPEs比母体OPEs具有更高的生物毒性^［6］^，如Su等^［7］^证实磷酸三苯酯（TPHP）的代谢产物磷酸二苯酯（DPHP）可以诱导更多基因的异常表达，Xu等^［8］^证实小鼠体内mOPEs的残留水平通常高于OPEs。PAEs是一类广泛使用的高产工业化学品，低相对分子质量PAEs（主链3～6碳原子）普遍用于医疗器械、食品包装、玩具和个人护理产品等领域；高相对分子质量PAEs（主链7～13碳原子）主要用作增塑剂，尤其是添加到聚氯乙烯塑料中以增强其延展性和适应性，广泛应用于电线电缆、地板、涂料等^［9，10］^。PAEs是一种抗雄激素干扰物，在动物实验中显示有致畸、致癌作用，同时会影响生殖发育等生理过程，能导致精子质量下降、儿童性早熟，对神经系统、生殖系统和内分泌系统的影响均有报道^［2，11，12］^。PAEs的主要代谢产物（mPAEs）是邻苯二甲酸单酯。通常，新陈代谢的第一步是解毒，但体外和体内实验均表明，邻苯二甲酸单酯具有更强的生物活性，对人体危害也更大^［9，13］^。此外，PAEs广泛存在于实验室环境和实验耗材中，现场空白浓度可能与收集的样本中的浓度相当^［14］^，PAEs在人体和啮齿动物的血浆或尿液中的半衰期均不足24 h，多项研究对PAEs本体的药代动力学进行了综述，发现PAEs本体在胃肠道中会快速水解为mPAEs，单纯检测PAEs本体很难抓住这个窗口期，而以分析mPAEs代替PAEs的分析可以克服这一限制，故本研究选择mPAEs作为目标化合物。

OPEs和PAEs均是通过物理添加方式与多种日常消费品结合，在产品使用过程中易通过挥发、浸出或磨损释放到不同的环境介质中，目前在室内外空气、粉尘、水、土壤、农作物和生物材料中都检出了不同浓度的OPEs和PAEs^［15］^。由于OPEs和PAEs无处不在，在日常生活中人们往往会同时暴露于多种OPEs和PAEs，考虑到混合暴露的累加效应，即使单个物质的暴露量低于规定剂量，多种化合物的累积暴露也可能影响人类健康^［16］^。目前国内外对于OPEs和PAEs的研究主要集中在粉尘^［17，18］^、空气^［19，20］^、水体^［21，22］^和土壤^［23］^等外部暴露，对于人体内暴露评估较少，且主要集中于尿液的检测，鉴于OPEs和PAEs的广泛应用和繁多的暴露途径，内部暴露评估可以提供一个更真实的人类暴露情况，血液中OPEs和PAEs水平可能比尿液水平更能揭示人类长期暴露程度。此外，现有报道多为OPEs或PAEs的单一类别检测，OPEs和PAEs同时检测尚属罕见。

关于 OPEs 和 PAEs 的内暴露研究，到目前为止，多集中于尿液基质，而针对其在人体血液中的直接检测和报道相对较少。Wang等^［24］^采用液-液萃取（LLE）结合固相萃取（SPE）柱从1 mL全血中提取OPEs，采用超高效液相色谱-串联质谱（UPLC-MS/MS）测定OPEs。Liu等^［25］^开发了一种从500 μL血清中提取OPEs的SPE预处理方法。SPE、LLE和蛋白沉淀法（PPT）是LC-MS/MS常用的预处理方法。常规的SPE法在样品预处理过程中烦琐耗时且实验成本高，限制了其在大样本人群生物监测中的应用，复杂的处理工艺容易增加实验的不确定性，影响测定结果的稳定性和准确性。LLE是传统的前处理方法，方便快捷，但其缺点是消耗大量有机溶剂，且需要样品量较大，不能满足于血液样品的常规检测。PPT是一种广泛应用于生物样品的净化阳离子和提取的方法，具有简单、成本低、易于实现等优点，但其容易带来基质效应。HMR S-micro BIO 96孔固相萃取板是一种新型的固相萃取材料，其为滤过式SPE，简化了常规SPE活化、淋洗、洗脱等步骤，过程简单，快速。同时，高比表面积的聚合物骨架可以有效吸附拦截血液样品中的磷脂和大分子，去除基质干扰。

基于此，本研究将高通量的HMR S-micro BIO 96孔固相萃取板与UPLC-MS/MS技术结合，建立了一种可同时测定人体全血中22种OPEs、mOPEs和11种mPAEs的方法，为全面评估人体内新型污染物OPEs和PAEs的暴露情况与健康风险提供方法学支持。

## 1 实验部分

### 1.1 仪器、试剂与材料

岛津8060NX超高效液相色谱-三重四极杆质谱仪（日本岛津公司）；固相萃取正压装置、96孔氮吹仪、HMR S-micro BIO 96孔固相萃取板（20 mg，北京纳鸥科技有限公司）；5804R冷冻离心机（德国Eppendorf公司）；Multi Reax涡旋混匀器（德国Heidolph公司）；甲醇、乙腈和超纯水均为LC-MS级（德国默克公司）；甲酸（LC-MS级，美国阿拉丁公司）；乙酸铵（LC-MS级，北京百灵威科技有限公司）。

14种OPEs标准品和10种OPEs内标质量浓度均为100.0 mg/L，购自上海安谱璀世标准科技服务有限公司；8种mOPEs标准品（纯度94%~98%）和4种mOPEs内标（纯度95%~98%）购自Toronto Research Chemicals；11种mPAEs标准品（纯度95%~100%）和11种mPAEs内标（纯度95%~98%）购自Cambrideg Isotope Laboratories。目标化合物及同位素内标信息详见表1。

**表1:** 33种目标化合物及25种同位素内标的基本信息

Compound	Abbr.	CAS No.	Formula	*M*_r_
Organic phosphate esters （OPEs， 有机磷酸酯）				
Trimethyl phosphate （磷酸三甲酯）	TMP	512-56-1	C_3_H_9_O_4_P	140.07
Tris-（2-chloroethyl） phosphate （磷酸三（2-氯乙基）酯）	TCEP	115-96-8	C_6_H_12_Cl_3_O_4_P	285.49
Triisopropyl phosphate （磷酸三异丙酯）	TiPP	513-02-0	C_9_H_21_O_4_P	224.23
Tripropyl phosphate （磷酸三丙酯）	TnPP	513-08-6	C_9_H_21_O_4_P	224.23
2，2-Bis（chloromethyl） trimethylene bis（bis（2- chloroethyl）phosphate （磷酸2，2-双氯甲基-三亚甲基-双（双（2-氯乙基））酯）	V6	38051-10-4	C_13_H_24_Cl_6_O_8_P_2_	582.99
Tris-（2-chloroisopropyl）phosphate （磷酸三（1-氯-2-丙基）酯）	TCiPP	13674-84-5	C_9_H_18_Cl_3_O_4_P	327.57
Tris（2-chloro-1-（chloromethyl）ethyl）phosphate （磷酸三（1，3-二氯-2-丙基）酯）	TDCiPP	13674-87-8	C_9_H_15_Cl_6_O_4_P	430.90
Tris（2，3-dibromopropyl） phosphate （三-（2，3-二溴丙基）-磷酸酯）	TDBPP	126-72-7	C_9_H_15_Br_6_O_4_P	697.61
Triphenyl phosphate （磷酸三苯酯）	TPHP	115-86-6	C_18_H_15_O_4_P	326.28
Triisobutyl phosphate （磷酸三异丁酯）	TiBP	126-71-6	C_12_H_27_O_4_P	266.31
Tributyl phosphate （磷酸三丁酯）	TnBP	126-73-8	C_12_H_27_O_4_P	266.31
Tris-（2-butoxyethyl） phosphate（磷酸三（2-丁氧基乙基）酯）	TBOEP	78-51-3	C_18_H_39_O_7_P	398.47
Tricresyl phosphate （磷酸三间甲苯酯）	TMPP	1330-78-5	C_21_H_21_O_4_P	368.36
2-Ethylhexyl diphenyl phosphate （磷酸2-乙基己基二苯基酯）	EHDPP	1241-94-7	C_20_H_27_O_4_P	362.41
Organic phosphate esters of metabolites （mOPEs， 有机磷酸酯代谢物）				
Bis（2-butoxyethyl）2-hydroxyethyl phosphate triester （磷酸双（2-丁氧基乙基）2-羟基乙基三酯）	BBOEHEP	1477494-86-2	C_14_H_31_O_7_P	342.37
Bis（2-butoxyethyl）2-（3-hydroxyethyl）ethyl phosphate triester （双（2-丁氧基乙基）2-（3-羟基丁氧基）乙基磷酸酯）	OH-TBOEP	1477494-87-3	C_18_H_39_O_8_P	414.47
Bis（2-chloroethyl） hydrogen phosphate （磷酸氢二（2-氯乙基）酯）	BCEP	3040-56-0	C_4_H_9_Cl_2_O_4_P	222.99
Dibutyl phosphate （磷酸二正丁酯）	DNBP	107-66-4	C_8_H_19_O_4_P	210.21
Diphenyl phosphate （磷酸二苯酯）	DPHP	838-85-7	C_12_H_11_O_4_P	250.19
Bis（2-ethylhexyl） phosphate （磷酸二（2-乙基己基）酯）	BEHP	298-07-7	C_16_H_35_O_4_P	322.42
Bis（1-chloro-2-propyl） phosphate （磷酸二（2-氯丙基）酯）	BCiPP	789440-10-4	C_12_H_26_Cl_4_O_8_P_2_	502.08
Bis（butoxyethyl phosphate （磷酸二（2-丁氧基乙基）酯）	BBOEP	14260-97-0	C_12_H_27_O_6_P	298.31
Phthalate ester metabolites （mPAEs， 邻苯二甲酸酯代谢物）				
Monobenzyl phthalate （邻苯二甲酸单苄酯）	MBZP	2528-16-7	C_15_H_12_O_4_	256.25
Monocyclohexyl phthalate （邻苯二甲酸单环己酯）	MCHP	7517-36-4	C_14_H_16_O_4_	248.27
Monoisodecyl phthalate （邻苯二甲酸单异癸酯）	MDP	69725-01-5	C_18_H_26_O_4_	306.42
Mono（2-ethyl-5-hydroxyhexyl） phthalate （邻苯二甲酸单（2-乙基-5-羟己基）酯）	MEHHP	40321-99-1	C_16_H_22_O_5_	292.327
Mono（2-ethylhexyl） phthalate （邻苯二甲酸单（2-乙基己基）酯）	MEHP	4376-20-9	C_16_H_22_O_4_	278.3
Mono（2-ethyl-5-oxohexyl） phthalate （邻苯二甲酸单（2-乙基-5-氧己基）酯）	MEOHP	40321-98-0	C_16_H_20_O_5_	292.33
Monoethyl phthalate （邻苯二甲酸单乙酯）	MEP	2306-33-4	C_10_H_10_O_4_	194.18
Monomethyl phthalate （邻苯二甲酸单甲酯）	MMP	4376-18-5	C_9_H_8_O_4_	180.16
Mono-*n*-butyl phthalate （邻苯二甲酸单正丁酯）	MNBP	131-70-4	C_12_H_14_O_4_	222.24
Monoisononyl phthalate （邻苯二甲酸单异壬酯）	MNP	297182-83-3	C_17_H_24_O_4_	292.4
Mono-*n*-octyl phthalate （邻苯二甲酸单正辛酯）	MOP	5393-19-1	C_16_H_22_O_4_	278.3
IS				
TMP-D_9_ （磷酸三甲酯-D_9_）	TMP-D_9_	32176-12-8	C_3_D_9_O_4_P	149.13
TnPP-D_21_ （磷酸三丙酯-D_21_）	TnPP-D_21_	1219794-92-9	C_9_D_21_O_4_P	245.36
TnBP-D_27_ （磷酸三丁酯-D_27_）	TnBP-D_27_	61196-26-7	C_12_D_27_O_4_P	293.48
TPHP-D_15_ （磷酸三苯酯-D_15_）	TPHP-D_15_	1173020-30-8	C_18_D_15_O_4_P	341.38
TCEP-D_12_ （磷酸三（2-氯乙基）酯-D_12_）	TCEP-D_12_	1276500-47-0	C_6_D_12_Cl_3_O_4_P	297.56
TCiPP-D_18_ （磷酸三（2-氯丙基）酯-D_18_）	TCiPP-D_18_	1447569-78-9	C_9_D_18_Cl_3_O_4_P	345.68
TDCPP-D_15_ （磷酸三（1，3-二氯-2-丙基）酯-D_15_）	TDCPP-D_15_	1447569-77-8	C_9_D_15_Cl_6_O_4_P	446.00
TBOEP-D_27_ （三（2-丁氧基乙基）磷酸酯-D_27_）	TBOEP-D_27_	78-51-3-D_27_	C_18_H_12_D_27_O_7_P	425.64
V6-D_16_ （2，2-双氯甲基-三亚甲基-双［双（2-氯乙基）］磷酸酯-D_16_）	V6-D_16_	38051-10-4-D_16_	C_13_H_8_D_16_Cl_6_O_8_P_2_	599.09
TDBPP-D_15_ （三-（2，3-二溴丙基）-磷酸酯-D_15_）	TDBPP-D_15_	157801-77-9	C_9_D_15_Br_6_O_4_P	712.7
BBOEHEP-D_4_ （双（2-丁氧基乙基）2-羟基乙基磷酸三酯-D_4_）	BBOEHEP-D_4_	2469195-92-2	C_14_H_27_D_4_O_7_P	346.39
BCEP-D_8_ （磷酸二（2-氯乙基）酯-D_8_）	BCEP-D_8_	1477495-02-5	C_4_HD_8_Cl_2_O_4_P	231.04
DPHP-D_10_ （磷酸二苯酯-D_10_）	DPHP-D_10_	1477494-97-5	C_12_HD_10_O_4_P	260.25
BBOEP-D_8_ （磷酸二（2-丁氧基乙基）酯-D_8_）	BBOEP-D_8_		C_12_H_19_D_8_O_6_P	306.36
MBZP-^13^C_2_ （邻苯二甲酸单苄酯-^13^C_2_）	MBZP-^13^C_2_	2528-16-7	C_11_C_4_H_12_O_4_	260.22
MCHP-^13^C_2_（邻苯二甲酸单环己酯-^13^C_2_）	MCHP-^13^C_2_	7517-36-4	C_11_C_4_H_16_O_4_	252.25
MDP-D_4_ （邻苯二甲酸单异癸酯-D_4_）	MDP-D_4_		C_18_H_22_D_4_O_4_	310.42
MEHHP-^13^C_4_ （邻苯二甲酸单（2-乙基-5-羟己基）酯-^13^C_4_）	MEHHP-^13^C_4_	40321-99-1	C_12_C_4_H_22_O_5_	298.31
MEHP-^13^C_2_（邻苯二甲酸单（2-乙基己基）酯-^13^C_2_）	MEHP-^13^C_2_	959266-61-6	C_12_C_4_H_22_O_4_	282.31
MEOHP-^13^C_4_ （邻苯二甲酸单（2-乙基-5-氧己基）酯-^13^C_4_）	MEOHP-^13^C_4_	40321-98-0	C_12_C_4_H_20_O_5_	296.30
MEP-^13^C_2_（邻苯二甲酸单乙酯-^13^C_2_）	MEP-^13^C_2_	2306-33-4	C_6_C_4_H_10_O_4_	198.16
MMP-^13^C_2_ （邻苯二甲酸单甲酯-^13^C_2_）	MMP-^13^C_2_	4379-18-5	C_5_C_4_H_8_O_4_	184.13
MNBP-^13^C_2_ （邻苯二甲酸单正丁酯-^13^C_2_）	MNBP-^13^C_2_	131-70-4	C_8_C_4_H_14_O_4_	226.21
MNP-D_4_ （邻苯二甲酸单异壬酯-D_4_）	MNP-D_4_		C_17_H_20_D_4_O_4_	296.21
MOP-^13^C_2_ （邻苯二甲酸单正辛酯-^13^C_2_）	MOP-^13^C_2_	5393.19-1	C_12_C_4_H_22_O_4_	282.31

本研究获得常州疾病预防控制中心医学伦理委员会批准（批准文件编号：常疾控伦审［2024］09号）。样品采集：采用含抗凝剂（EDTA-K_2_）的采血管采集血液，轻柔颠倒采血管6~8次混匀后，转移至10 mL玻璃离心管中，于-80 ℃条件下保存。

### 1.2 标准溶液的配制

混合标准储备液：准确称取33种目标化合物标准品于100 mL容量瓶中，用甲醇溶解并定容，配制成质量浓度为100 ng/mL的混合标准储备液，于-20 ℃冰箱保存。

混合内标储备溶液：准确称取25种内标标准品于100 mL容量瓶中，用甲醇溶解并定容，配制成质量浓度为100 ng/mL的混合内标储备液，于-20 ℃冰箱保存。

混合标准工作液：使用时，准确吸取不同体积的混合标准储备液和1.0 mL混合内标储备溶液，用0.1%甲酸乙腈定容至10.0 mL配制成33种目标化合物质量浓度为0.1、0.2、0.5、1.0、2.0、5.0、10、50 ng/mL，25种同位素内标质量浓度为10 ng/mL的标准工作溶液。

### 1.3 样品前处理

将全血样品从-80 ℃冰箱中取出，置于室温下解冻，解冻后，使用玻璃枪头准确吸取200 μL全血于10 mL玻璃离心管中，加入100 μL混合内标储备溶液，加入2.0 mL 0.1%甲酸乙腈提取，涡旋混匀10 min，超声20 min，5 000 r/min离心5 min，将96孔固相萃取板用2 mL/孔的乙腈加压冲洗，取全部上清液过柱，采用正压装置施加压力，使其以1~2 mL/min的流速通过固相萃取柱，待管内液体全部流出，沿管壁继续加入1 mL乙腈，加压待全部液体流出；将洗脱液在40 ℃下氮吹至近干，加入100 μL 0.1%甲酸乙腈复溶，过0.22 μm PTFE滤头，转移至进样瓶（带玻璃内衬管），待测定。

设置0.1%甲酸乙腈复溶液为过程空白，健康人群全血样品通过上述前处理后设置为全血空白。

### 1.4 仪器分析条件

#### 1.4.1 液相色谱条件

将Ghost Trap DS-HP色谱柱（30 mm×2.1 mm）连接在混合器和进样器之间作为背景鬼峰捕集柱；以ACQUITY UPLC^®^ BEH C18色谱柱（150 mm×2.1 mm，1.7 μm，美国Waters公司）为分析柱；流动相：A为0.5 mmol/L乙酸铵水溶液，B为甲醇；梯度洗脱程序：0～2.0 min，5%B；2.0～2.1 min，5%B~50%B；2.1～9.0 min，50%B～98%B；9.0～12.0 min，98%B；12.0~13.0 min，98%B~5%B；13.0~15.0 min，5%B；流速：0.30 mL/min；柱温：40 ℃；进样体积：5 μL。

#### 1.4.2 质谱条件

电喷雾电离源离子模式（ESI^+^/ESI^-^）；多反应监测（MRM）模式采集；雾化气流量：3 L/min，加热气流量：15 L/min，接口温度：400 ℃，脱溶剂管温度：250 ℃，脱溶剂温度：650 ℃，加热块温度：400 ℃。化合物质谱参数见表2。

**表2:** 33种目标化合物及25种内标的质谱参数

Compound	Retention time/min	Precursor ion （*m/z*）	Product ions （*m/z*）	Q1/V	CE/V	Q3/V	IS
TMP	4.51	141.1	109.0^*^， 79.0	-21	-19	-20	TMP-D_9_
TCEP	5.92	285.1	63.0^*^， 99.0	-17	-27	-25	TCEP-D_12_
TiPP	7.25	225.1	99.0^*^， 141.0	-14	-20	-18	TnPP-D_21_
TnPP	6.95	225.1	99.0^*^， 141.0	-14	-20	-18	TnPP-D_21_
V6	7.31	582.9	361.0^*^， 297.0	-38	-19	-25	V6-D_16_
TCiPP	7.26	327.1	99.0^*^	-20	-21	-18	TCiPP-D_18_
TDCiPP	8.28	431.1	99.0^*^， 209.0	-26	-27	-18	TDCPP-D_15_
TDBPP	8.71	698.6	99.0^*^， 298.9	-24	-28	-18	TDBPP-D_15_
TPHP	8.39	327.1	77.0^*^， 215.1	-20	-41	-14	TPHP-D_15_
TiBP	9.03	267.2	99.0^*^， 155.0	-17	-20	-18	TnBP-D_27_
TnBP	8.90	267.2	99.0^*^， 155.0	-17	-21	-17	TnBP-D_27_
TBOEP	9.33	399.4	199.1^*^， 299.2	-24	-17	-21	TBOEP-D_27_
TMPP	9.97	369.4	91.0^*^， 166.1	-22	-39	-17	TPHP-D_15_
EHDPP	10.33	385.2	273.0^*^	-24	-16	-19	TPHP-D_15_
BBOEHEP	7.14	343.0	243.0^*^， 45.0	-23	-12	-17	BBOEHEP-D_4_
OH-TBOEP	7.79	415.0	45.0^*^， 199.0	-20	-13	-15	TBOEP-D_27_
BCEP	4.37	223.0	99.1^*^， 62.9	-15	-16	-24	BCEP-D_8_
DNBP	5.38	209.1	79.0^*^， 153.0	20	23	13	DPHP-D_10_
DPHP	5.09	249.0	93.0^*^， 155.0	22	30	30	DPHP-D_10_
BEHP	8.60	321.0	79.0^*^， 209.2	12	29	13	TPHP-D_15_
BCiPP	5.08	249.0	93.0^*^， 154.7	21	28	15	DPHP-D_10_
BBOEP	5.86	297.0	79.0^*^， 197.2	22	25	12	BBOEP-D_8_
MBZP	5.02	255.0	77.0^*^， 183.0	23	21	30	MBZP-^13^C_2_
MCHP	5.43	247.1	77.0^*^， 97.1	23	19	28	MCHP-^13^C_2_
MDP	7.85	305.1	154.9^*^， 77.0	28	24	30	MDP-D_4_
MEHHP	5.26	293.0	121.0^*^， 145.0	24	16	12	MEHHP-^13^C_4_
MEHP	6.70	276.9	134.0^*^， 77.0	20	14	21	MEHP-^13^C_2_
MEOHP	4.96	291.0	121.0^*^， 143.0	30	16	21	MEOHP-^13^C_4_
MEP	4.37	192.9	77.0^*^， 121.0	14	17	11	MEP-^13^C_2_
MMP	3.31	179.1	77.0^*^， 107.0	15	16	12	MMP-^13^C_2_
MNBP	5.02	221.1	76.9^*^， 71.0	21	17	28	MNBP-^13^C_2_
MNP	6.95	291.1	140.9^*^， 77.0	13	26	11	MNP-D_4_
MOP	6.90	277.1	77.0^*^， 127.0	22	22	11	MOP-^13^C_2_
TMP-D_9_	4.49	150.1	115.0	-26	-20	-21	/
TnPP-D_21_	7.16	246.1	102.0	-16	-20	-19	/
TnBP-D_27_	8.93	294.2	102.0	-21	-22	-19	/
TPHP-D_15_	8.31	342.1	82.1	-22	-46	-15	/
TCEP-D_12_	5.88	297.1	102.1	-18	-23	-19	/
TCiPP-D_18_	7.19	345.1	102.0	-21	-22	-19	/
TDCPP-D_15_	8.23	446.1	102.0	-28	-28	-18	/
TBOEP-D_27_	9.25	426.4	208.0	-14	-17	-22	/
V6-D_16_	7.32	598.9	369.0	-38	-20	-26	/
TDBPP-D_15_	8.65	713.6	102.0	-24	-28	-18	/
BBOEHEP-D_4_	7.14	347.0	247.0	-23	-12	-17	/
BCEP-D_8_	4.31	231.0	67.1	-16	-21	-27	/
DPHP-D_10_	5.02	259.0	98.0	23	19	28	/
BBOEP-D_8_	5.90	305.0	79.0	22	27	12	/
MBZP-^13^C_2_	4.96	259.0	76.9	13	17	12	/
MCHP-^13^C_2_	5.20	251.1	79.0	13	17	13	/
MDP-D_4_	6.72	309.1	81.2	19	14	13	/
MEHHP-^13^C_4_	5.08	297.1	124.2	20	22	12	/
MEHP-^13^C_2_	5.26	281.0	136.9	20	22	12	/
MEOHP-^13^C_4_	3.37	295.2	123.9	13	27	12	/
MEP-^13^C_2_	4.55	197.0	79.0	15	17	12	/
MMP-^13^C_2_	7.80	183.0	79.0	24	23	13	/
MNBP-^13^C_2_	6.96	225.0	79.0	13	27	12	/
MNP-D_4_	4.96	295.1	79.2	18	17	12	/
MOP-^13^C_2_	6.91	280.9	127.0	10	15	13	/

* Quantitative ion pair； /： no value.

## 2 结果与讨论

### 2.1 色谱条件优化

本研究考察了3种色谱柱：ACQUITY UPLC^®^ BEH C18（100 mm×2.1 mm，1.7 μm）、ACQUITY UPLC^®^ BEH C18（150 mm×2.1 mm，1.7 μm）和Kinetex F5（100 mm×3.0 mm，2.6 μm）对33种目标化合物的分离效果。实验表明，使用100 mm BEH C18色谱柱时，MEHP和MOP这对同分异构体未能实现完全分离；150 mm的BEH C18色谱柱在相同仪器条件下，33种目标化合物均具有较满意的峰形与响应，MEHP和MOP这对同分异构实现完全分离，且在15 min内33种目标化合物可全部出峰（见图1）；使用Kinetex F5柱时，OH-TBOEP、BBOEP、MEOHP等化合物的色谱峰出现峰形拖尾，且mPAEs的响应普遍比BEH C18色谱柱降低50%，同时，由于Kinetex F5柱的偶极和*π-π*作用，目标化合物分析时间延长至25 min。因此选择ACQUITY UPLC^®^ BEH C18（150 mm×2.1 mm，1.7 μm）色谱柱作为分析柱。

**图1:**
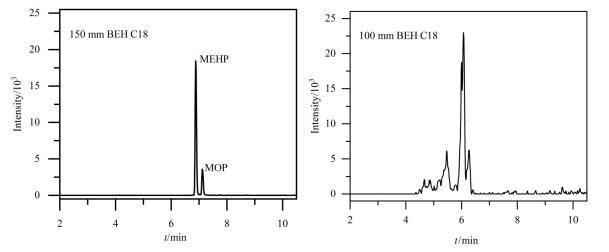
不同长度BEH C18色谱柱对MEHP和MOP的分离效果

对流动相进行优化，在超纯水作为水相的条件下，所有33种目标化合物在甲醇中的响应值均高于乙腈，尤其对于大多数OPEs和mOPEs影响较大，故本研究选择甲醇作为流动相。进一步对水相进行优化，选择0.5 mmol/L乙酸铵水溶液和0.1%乙酸水溶液两种流动相，结果表明，当水相为0.5 mmol/L乙酸铵水溶液时，多数OPEs和mOPEs的响应比0.1%乙酸水溶液高3~4倍，多数mPAEs的响应比0.1%乙酸水溶液降低10%~20%，说明0.1%的乙酸对多数OPEs和mOPEs的电离有抑制作用，对mPAEs有促进作用。为提高mPAEs的响应且不影响OPEs和mOPEs的响应，选择向0.5 mmol/L乙酸铵中加入不同含量（0.1%、0.05%和0.02%）的乙酸，结果表明即使0.5 mmol/L乙酸铵中仅含有0.02%的乙酸，多数OPEs和mOPEs的响应仍然降低了50%~75%，且mPAEs响应仅提升10%~20%，故选择放弃加入乙酸。继续比较不同乙酸铵浓度（0.05、0.1、0.5、1.0 mmol/L）对化合物响应的影响，结果表明0.05和0.1 mmol/L的乙酸铵会降低OPEs和mOPEs的响应，对mPAEs响应影响不明显，1.0 mmol/L的乙酸铵对目标化合物响应影响不明显，但会显著增大基线噪声，影响方法的灵敏度，综合以上结果，本研究最终选择0.5 mmol/L乙酸铵-甲醇作为流动相。在1.4节条件下，33种目标化合物的总离子流图见图2，可以看出，33种目标化合物的色谱峰形和响应强度均较为理想。

**图2:**
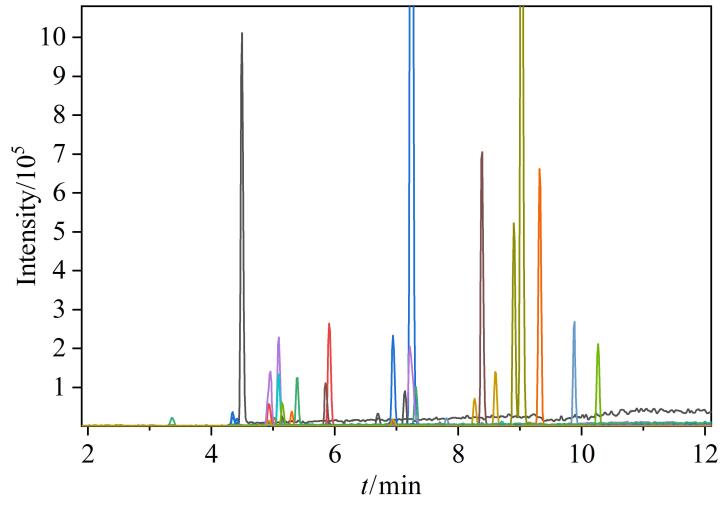
33种目标化合物的总离子流图

### 2.2 前处理条件的优化

#### 2.2.1 提取溶剂

实验比较了不同溶剂的提取效率，通过在全血样品中加标20 ng/mL（终质量浓度），分别采用5种提取溶剂（乙腈、甲醇、乙腈-甲醇（1∶1）、0.1%甲酸乙腈、0.5%甲酸乙腈）进行提取，不经过固相萃取柱处理，其余按照1.3节处理，计算回收率。采用乙腈为提取溶剂时，TCEP、MEP、MMP、BBOEP的回收不理想，回收率分别为204.62%、13.24%、197.83%、159.95%；采用甲醇提取时，TMP和BCEP未出峰，OH-TBOEP的回收达到187.15%；提取溶液为乙腈-甲醇（1∶1）时，TMP和BCEP未出峰，DPHP回收率低于10%；提取溶液为0.1%甲酸乙腈时，33种目标化合物的回收率均在60.48%~137.37%；提取溶液为0.5%甲酸乙腈时，MEOHP的回收率为188.86%，MMP-^13^C_2_未出峰。此外，当提取溶液中存在乙腈时，形成的沉淀为大粒径固体沉淀物，甲醇则形成松散的絮凝沉淀物，离心后，其絮状沉淀物易悬浮。综上，本研究最终选择0.1%甲酸乙腈为提取溶剂。

#### 2.2.2 固相萃取柱

考察了3种固相萃取柱：HMR S-micro BIO 96孔SPE（A）、Anavo HLB-Prime SPE （6 mL，300 mg）（玻璃）（B）和Oasis HLB-Prime SPE （3 mL，150 mg）（塑料）（C）。在全血样品中加标20 ng/mL（终质量浓度），按照1.3节操作，计算目标化合物的回收率。结果表明，选择A时，33种目标化合物的回收率为67.89%~130.14%；选择B时，TMP、TDBPP、BCEP未出峰，TnPP的回收率为20.63%，DNBP、BEHP的回收率达到200%；选择C时，TnPP的回收率为23.92%，OH-TBOEP的回收率为173.24%，BCEP未出峰。由结果可知，HMR S-micro BIO 96孔柱满足“生物样品定量分析方法验证指导原则”的要求，最终选择HMR S-micro BIO 96孔SPE。

TnPP在2.2.1节中使用不同提取溶剂提取但不经过SPE净化，其回收率在77.46%~125.51%，经过HLB柱净化后其回收率均显著降低，但是其同分异构体TiPP未出现此情况，可能的原因是TnPP是直链结构，空间位阻小，其分子结构中P=O键易与HLB柱硅胶表面的硅羟基发生相互作用，继而影响其回收率，而HMR S-micro BIO 96孔SPE小柱填料为无机聚合物，可避免TnPP的损失。

#### 2.2.3 提取液体积

考察了不同体积（0.2、0.4、1.0、2.0、3.0 mL）0.1%甲酸乙腈提取溶剂对实验的影响。在全血样品中加标20 ng/mL（终质量浓度），按照1.3节操作，计算目标化合物的回收率。当提取溶剂过少时（0.2、0.4 和1.0 mL），全血样品中的蛋白质等大分子物质未沉淀完全，会堵塞SPE柱，在后续仪器检测过程中影响基线平衡，导致噪声增大；当提取溶剂为3 mL时，由于沉淀速度过快，与高血浆蛋白结合的分析物会发生共沉淀，影响回收率，如mPAEs回收率普遍偏低（<60%）。故本研究最终选择提取液体积为2.0 mL。

#### 2.2.4 污染控制

预实验结果表明，TCEP、TCiPP、TPHP、TiBP和TnBP存在较明显的背景干扰。文献［^26^］报道，在混合器与自动进样器之间连接捕集柱可有效降低流动相中的背景干扰。基于此，本研究选用了Ghost Trap DS-HP色谱柱（30 mm×2.1 mm）作为捕集柱。在0.5 mmol/L乙酸铵-甲醇流动相条件下，安装捕集柱后，背景干扰中TCiPP的响应强度较未安装前下降94.7%，其余残留背景干扰亦得到有效消除。

前处理过程中易引入OPEs及mOPEs等背景干扰，因此需对所有试剂与耗材进行本底筛查。本实验所用试剂（包括超纯水）均选用质谱级，以最大程度降低试剂引入的背景干扰。由于塑料材质（包括聚丙烯）可能带来污染，建议实验过程中尽量避免使用。本实验除0.22 μm滤头为聚四氟乙烯（PTFE）材质外，其余耗材均采用玻璃材质，具体包括10 mL玻璃离心管、1 mL玻璃加样枪头、玻璃注射器、玻璃滴管、微量注射器及玻璃内衬管等。所有玻璃耗材在使用前均以锡箔纸包裹，置于400 ℃马弗炉中煅烧4 h，冷却后备用。该方法可有效降低TiBP和TnBP等目标物的污染风险。

对于实验过程中不可避免需使用的塑料制品：PTFE滤头和96孔SPE，均采用乙腈进行预处理以降低背景干扰。背景干扰检测结果表明：PTFE滤头经1 mL乙腈清洗一次后，可有效降低TiBP和TnBP含量；96孔SPE经2 mL乙腈清洗一次后，可显著降低TiBP、TnBP、MEHP和MNBP含量。

为控制仪器残留，本方法在每针进样前后均增加了洗针步骤，并将洗针液更换为强洗液体系（甲醇-乙腈-异丙醇-水，体积比为1∶1∶1∶1）。在高浓度样品进样后，额外进样0.1%甲酸乙腈以确保无残留。此外，每批样品分析过程中均穿插进样标准溶液，用于实时校正保留时间。

实验过程中发现，TMP加标全血样品在放置半个月后出现双峰现象，且响应强度下降64.6%。推测其原因可能是TMP相对分子质量较小、亲水性较强并具有一定挥发性，其酯键在储存过程中发生降解，生成磷酸二甲酯（DMP），从而导致色谱图中出现TMP与DMP两个色谱峰，见图3。因此，建议混合标准工作液现配现用，以保证分析结果的准确性。

**图3:**
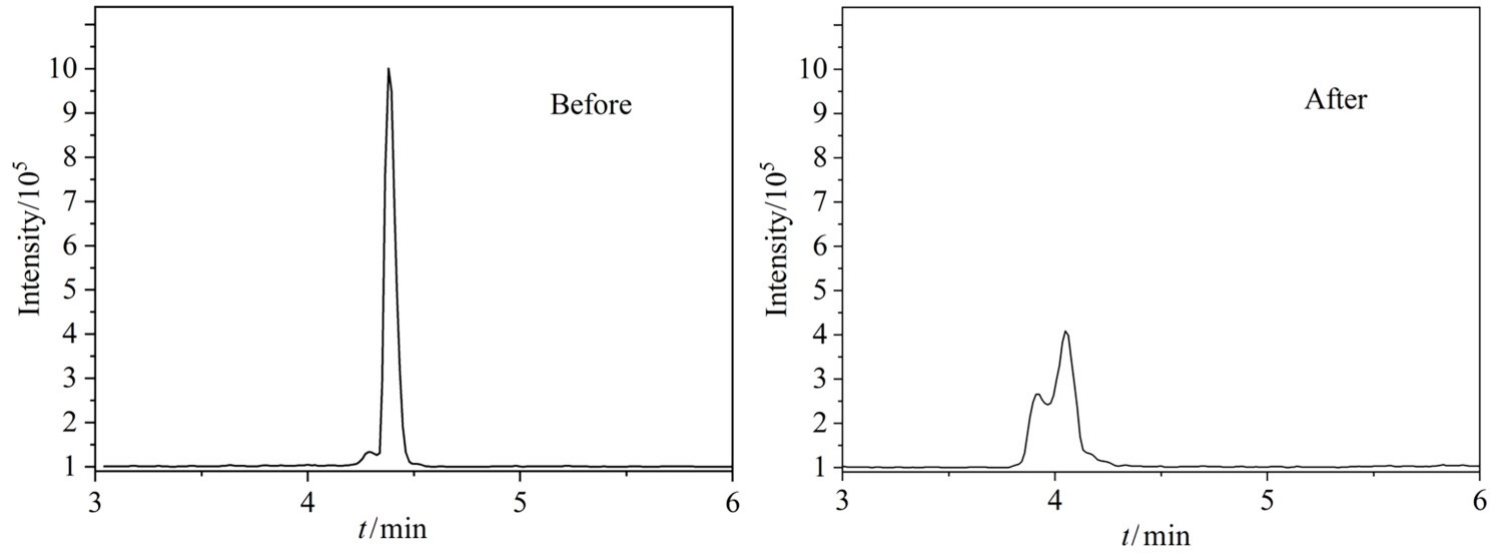
全血中TMP（1.0 ng/mL）放置15天前后的色谱图变化

### 2.3 方法学考察

#### 2.3.1 线性范围、方法检出限及定量限

采用本方法对系列质量浓度的混合标准工作液进行 LC-MS/MS 分析。以目标化合物质量浓度与对应同位素内标质量浓度之比为横坐标，目标化合物的峰面积与对应同位素内标峰面积之比为纵坐标，绘制标准曲线。结果显示，33种目标化合物线性关系良好，相关系数（*r*）为0.993 3~0.999 8。按照3倍信噪比（*S/N*）计算检出限（LOD），按照10倍*S/N*计算定量限（LOQ）。相关数据见表3。

**表3:** 33种目标化合物的线性回归方程、相关系数、检出限及定量限

Compound	Regression equation	*r*	LOD/（ng/mL）	LOQ/（ng/mL）
TMP	*Y*=0.37*X+*1.1160	0.9981	0.04	0.14
TCEP	*Y*=0.13*X*+0.2328	0.9998	0.03	0.09
TiPP	*Y*=0.16*X*+0.0144	0.9995	0.01	0.03
TnPP	*Y*=0.01*X*+0.0011	0.9998	0.17	0.56
V6	*Y*=0.43*X*+0.0123	0.9997	0.01	0.05
TCiPP	*Y*=0.27*X*+2.4939	0.9982	0.04	0.15
TDCiPP	*Y*=0.05*X-*0.00387	0.9995	0.02	0.07
TDBPP	*Y*=0.34*X*+0.0469	0.9985	0.03	0.11
TPHP	*Y*=0.15*X*+0.0472	0.9998	0.01	0.03
TiBP	*Y*=0.21*X-*0.0155	0.9993	0.01	0.02
TnBP	*Y*=0.02*X*+0.0058	0.9978	0.08	0.28
TBOEP	*Y*=0.31*X*+0.0028	0.9983	0.003	0.01
TMPP	*Y*=0.13*X-*0.0205	0.9988	0.01	0.04
EHDPP	*Y*=0.18*X-*0.0074	0.9990	0.01	0.02
BBOEHEP	*Y*=0.45*X-*0.0352	0.9995	0.01	0.03
OH-TBOEP	*Y*=0.096*X*+0.0017	0.9987	0.22	0.74
BCEP	*Y*=0.046*X*+0.0045	0.9998	0.31	1.02
DNBP	*Y*=0.03*X*+0.0055	0.9933	0.07	0.25
DPHP	*Y*=0.063*X*+0.0083	0.9989	0.05	0.16
BEHP	*Y*=0.028*X-*0.00049	0.9996	0.06	0.19
BCiPP	*Y*=0.05*X-*0.0014	0.9997	0.06	0.20
BBOEP	*Y*=0.093*X*+0.0023	0.9998	0.05	0.15
MBZP	*Y*=0.081*X*+0.0150	0.9976	0.15	0.50
MCHP	*Y*=0.094*X-*0.0047	0.9996	0.11	0.37
MDP	*Y*=0.038*X-*0.0061	0.9942	0.12	0.38
MEHHP	*Y*=0.11*X*+0.0066	0.9983	0.03	0.10
MEHP	*Y*=0.050*X*+0.02161	0.9986	0.02	0.06
MEOHP	*Y*=0.063*X-*0.0132	0.9968	0.10	0.35
MEP	*Y*=0.070*X*+0.0360	0.9951	0.09	0.30
MMP	*Y*=0.11*X*+0.1109	0.9980	0.03	0.11
MNBP	*Y*=0.48*X*+0.0381	0.9991	0.02	0.08
MNP	*Y*=0.059*X*+0.0064	0.9954	0.13	0.43
MOP	*Y*=0.092*X*+0.0113	0.9996	0.04	0.14

*Y*： peak area ratio of target compound to IS； *X*： mass concentration ratio of target compound to IS； linear range： 0.1-50 ng/mL.

#### 2.3.2 回收率与精密度

在空白全血样本中加入不同水平（5、20、40 ng/mL）的混合标准溶液，按1.3节和1.4节操作处理，以考察方法的准确度与精密度。每个加标水平设置6个平行样，同时设置过程空白和全血空白，进样6针即穿插检测过程空白和全血空白，以保证方法的准确性。结果表明，33种目标化合物的加标回收率为60.5%～138.3%，相对标准偏差（RSD）为1.6%～11.0%，见表4。

**表4:** 33种目标化合物的加标回收率和相对标准偏差（*n*=6）

Compound	5 ng/mL			20 ng/mL			40 ng/mL	
	Recovery/%	RSD/%		Recovery/%	RSD/%		Recovery/%	RSD/%
TMP	64.4	6.6		68.4	7.7		75.1	2.4
TCEP	109.7	2.6		104.1	5.7		118.5	10.1
TiPP	105.6	3.0		110.3	9.0		111.7	4.0
TnPP	77.5	7.8		83.8	6.4		104.8	6.5
V6	101.6	3.2		113.8	1.8		122.3	2.1
TCiPP	68.5	1.8		66.7	3.6		61.4	3.5
TDCiPP	118.7	5.4		104.5	7.5		94.1	7.3
TDBPP	85.1	9.1		92.6	5.6		107.3	5.9
TPHP	97.2	2.3		97.2	9.3		111.4	9.4
TiBP	71.2	5.6		81.4	2.9		80.3	2.7
TnBP	88.9	4.6		105.1	10.7		109.0	10.6
TBOEP	112.4	8.9		112.3	4.5		129.0	4.1
TMPP	86.0	6.7		81.4	8.0		91.5	8.8
EHDPP	83.9	11.0		77.7	6.9		93.3	6.8
BBOEHEP	88.9	1.6		105.4	1.9		79.9	2.4
OH-TBOEP	126.8	9.6		117.8	3.8		127.9	3.3
BCEP	110.5	7.2		138.3	7.4		103.6	7.1
DNBP	79.7	5.0		87.4	5.8		87.9	5.3
DPHP	120.6	4.0		73.6	3.9		68.5	9.2
BEHP	95.3	2.8		123.0	2.2		121.7	2.1
BCiPP	68.7	10.0		60.5	10.9		77.7	10.8
BBOEP	103.0	4.3		97.6	4.2		100.6	4.4
MBZP	97.5	8.6		91.7	5.4		97.1	2.7
MCHP	119.9	6.1		114.2	4.3		109.1	6.3
MDP	76.7	8.7		94.0	2.0		79.1	2.5
MEHHP	114.5	3.4		104.4	2.7		105.2	3.1
MEHP	93.6	7.9		111.3	4.3		121.8	7.0
MEOHP	118.6	5.2		129.9	2.8		128.3	5.5
MEP	84.3	3.8		78.2	4.2		123.8	5.9
MMP	89.9	2.5		97.0	2.6		112.1	2.0
MNBP	106.9	5.1		112.1	4.3		114.1	3.4
MNP	92.5	4.9		83.8	4.8		108.7	4.7
MOP	103.9	6.4		124.0	3.1		102.3	2.1

值得注意的是，部分化合物的回收率超出常规可接受范围（80%~120%），如TMP回收率偏低，原因可能是在前处理过程中部分降解为DMP；TCiPP回收率偏低的主要原因在于其疏水特性（log *P*约为1.5~2.0），易在进样系统中发生不可逆吸附。部分mPAEs如MEHP、MEOHP和MEP的高浓度加标回收率大于100%，这类单酯类化合物通常含有羧基，在负离子模式下其电离行为对微环境变化较为敏感，人全血共萃取的物质可能会加大MEHP、MEOHP和MEP的质子转移效率，导致其回收率偏高。然而，该方法在所有浓度水平下对这些化合物均展现出良好的精密度（RSD<11%），证明其用于定量的可靠性。TCEP、TnBP、EHDPP和BCiPP的RSD大于10%，其中，EHDPP和BCiPP未找到对应的同位素内标进行校正，而是分别用TPHP-D_15_和DPHP-D_10_作为同位素内标，这可能是其RSD大于10%的原因；TCEP和TnBP展现出良好的回收率（88.9%~118.5%）和相关性（*r*>0.993 3），证明了定量的可靠性。

### 2.4 实际样品的测定

为验证方法的实用性，收集14份人全血样本，按照本研究方法开展目标化合物检测。检测结果表明，在14份样本中TCiPP、TPHP、EHDPP、MEHHP、MNBP和MOP的检出率为100%，TPHP的检出量最高（10.04 ng/mL），具体见表5。Guo等^［27］^研究表明，在越南河内、归洲和日本神奈川地区的母乳中TPHP是首要成分。Sundkvist等^［28］^在瑞典母乳样本中检测到TCiPP是OPEs污染主要成分。MEHHP是邻苯二甲酸二-2-乙基己酯（DEHP）的代谢产物之一，DEHP作为商业上主要使用的邻苯二甲酸酯^［29］^，主要应用于聚氯乙烯。本研究中MEHHP、MNBP的中位数质量浓度分别为 0.15和 0.24 ng/mL，与Högberg等^［29］^对瑞典36名女性血清的检测结果（MEHHP、MNBP中位数质量浓度均为0.5 ng/mL）类似。

**表5:** 人体全血样本中目标化合物的测定结果（*n*=14）

Compound	Detection rate/%	Median/（ng/mL）	Maximum/（ng/mL）	IQR/（ng/mL）
TMP	-	-	-	-
TCEP	-	-	-	-
TiPP	85.7	0.42	0.67	0.21
TnPP	-	-	-	-
V6	-	-	-	-
TCiPP	100	0.57	0.95	0.24
TDCiPP	71.4	0.29	2.02	0.54
TDBPP	-	-	-	-
TPHP	100	2.14	10.04	6.07
TiBP	-	-	-	-
TnBP	14.3	-	0.55	0.23
TBOEP	50	1.44	1.99	0.58
TMPP	-	-	-	-
EHDPP	100	0.13	0.57	0.15
BBOEHEP	-	-	-	-
OH-TBOEP	-	-	-	-
BCEP	92.9	0.2	0.72	0.14
DNBP	14.3	-	0.58	0.11
DPHP	92.9	0.051	0.43	0.30
BEHP	28.6	-	1.74	0.39
BCiPP	-	-	-	-
BBOEP	71.4	0.28	0.89	0.3
MBZP	-	-	-	-
MCHP	78.6	0.62	1.86	0.76
MDP	-	-	-	-
MEHHP	100	0.15	0.75	0.21
MEHP	92.9	0.62	1.77	0.6
MEOHP	92.9	0.11	3.47	2.38
MEP	92.9	0.902	3.24	1.62
MMP	-	-	-	-
MNBP	100	0.24	0.32	0.38
MNP	78.6	0.21	1.03	0.87
MOP	100	0.52	0.73	0.13

-： <LOD； IQR： interquartile range.

同时，尿液作为OPEs和PAEs的首选样本，虽然易受饮食、代谢等方面的影响，但因其无损伤采样，仍是人体生物监测研究的首选样本。邻苯二甲酸二异辛酯（DEP）来自化妆品和个人护理产品，是人体接触的最主要的PAEs，DEP体内代谢产物为MEP，在本研究中MEP的检出率为92.9%。mPAEs在人体尿液中的浓度通常比脂质富集部位（如乳房组织等）高出5~20倍。例如，Högberg等^［29］^报道尿液中MEHP、MIBP、MEP和MNBP的浓度是血液或乳汁中的20~100倍。德国巴伐利亚北部地区的尿液样本显示，尿液中MBP、MEP、MEHHP和MEOHP的中位质量浓度分别为181、90.2、46.8和36.5 ng/mL^［31］^。Hu等^［32］^报道中国柳州青少年（6~18岁）尿液中TCEP、BCiPP和DPHP的中位质量浓度分别为0.28、0.35和0.24 ng/mL，高于本研究中全血的浓度。OPEs和PAEs对人类健康的潜在风险已成为学界关注的焦点。然而，关于其在环境中的存在状况、人体暴露途径及相应的健康风险，我们的认知尚不充分。本研究建立的分析方法可为准确评估人体对该类污染物的暴露水平及其健康风险提供可靠的技术支撑。

## 3 结论

本研究将高通量的HMR S-micro BIO 96孔固相萃取板与UPLC-MS/MS技术结合，建立了一种可同时测定人体全血中22种OPEs、mOPEs和11种mPAEs的方法。该方法具有操作简便、快速的特点，同时具备高灵敏度、良好的准确度与精密度，可应用于大批量实际全血样品的检测，能为全面评估人体内新型污染物OPEs和PAEs的暴露情况与健康风险提供方法学支持。
